# Complete genome sequences of two *Paenibacillus* isolated from pegmatite in Fukushima, Japan

**DOI:** 10.1128/mra.00938-24

**Published:** 2024-10-21

**Authors:** Phillip K. Yamamoto, Yuto Fujimoto, Tomoro Warashina, Kazuharu Arakawa

**Affiliations:** 1 Institute for Advanced Biosciences, Keio University, Tsuruoka, Yamagata, Japan; 2 Systems Biology Program, Graduate School of Media and Governance, Keio University, Fujisawa, Kanagawa, Japan; 3 Faculty of Environment and Information studies, Keio University, Fujisawa, Kanagawa, Japan; DOE Joint Genome Institute, Berkeley, California, USA

**Keywords:** *Paenibacillus*, Nanopore sequencing, complete genome, pegmatite

## Abstract

The genus *Paenibacillus* comprises facultatively anaerobic, endospore-forming, Gram-positive eubacteria known for efficiently producing a diverse array of exoenzymes. We isolated and sequenced the complete genomes of two *Paenibacillus* bacteria from the pegmatite surface in the fourth ore body in the mine Wagu Kannon Mine, Fukushima Prefecture.

## ANNOUNCEMENT

*Paenibacillus* is a genus of Gram-positive, facultatively anaerobic, endospore-forming bacteria with a rod shape, known for efficiently producing a diverse array of exoenzymes (1). We have isolated two *Paenibacillus* strains in 17th March 2023 from the surface of a pegmatite in the fourth ore body in the mine Wagu-kannon located in Fukushima, Japan (37.16400882685028, 140.41975314925645), which is characterized by large mineral crystals and high purity. Pegmatite samples were collected directly from the mine tunnel using sterilized chisels to avoid contamination.

We first isolated the microbes by suspending the crushed pegmatites in phosphate-buffered saline (PBS) and by applying to LB (WG01) or to R2A (WG02) agar medium incubated at 20°C. Pure colonies were obtained by performing the procedure of picking single colonies from the plates multiple times. Single colony was then picked and incubated in LB liquid medium for 48 h and stored as a glycerol stock. Subsequently, a single colony was again picked and incubated at 30°C for 10 h in LB liquid medium. Subsequently, genome DNA was extracted using NucleoBond HMW DNA kit (Macherey-Nagel, Düren, Germany) according to the manufacturer’s protocol. Long-read sequencing libraries were prepared using Rapid Barcoding Kit (SQK-RBK114.24, Oxford Nanopore Technologies) and sequenced on the GridION device using R10.4.1 Flowcell (FLO-MIN114, Oxford Nanopore Technologies). Sequenced reads were basecalled, demultiplexed, and trimmed for the adaptor sequences using Guppy 6.5.7 (super accurate mode). The quality of the long reads was assessed using Nanoplot (2), and reads longer than 10 kb (around x100 coverage) were adapter trimmed using Porechop v0.2.3 (3) and were then assembled using Canu version 2.2 (4). The assemblies generated a single assembled chromosome, which was manually circularized by deleting the overlapping ends. The completeness statistics saturated by a single round of error correction using medaka v.1.11.3 with the reads after adapter trimming. The completeness of the assembly was assessed with CheckM version 1.2.2 (5) to be 99.85% (WG01) and 99.55% (WG02) with automatic lineage identification (f__Paenibacillaceae (UID973)). Each circular genome was annotated using DDBJ Fast Annotation and Submission Tool (DFAST) version 1.2.18 (6) and was rotated so that *dnaA* gene was located at the start position. Sequence accession numbers, sequenced reads, genome assembly, and annotation statistics are listed in Table 1. Default parameters were used except where otherwise noted.

**TABLE 1:** Accession numbers and sequencing statistics

Bacterial species	*Paenibacillus polysaccharolyticus*	*Paenibacillus* sp.
Isolate	WG01	WG02
Accession no.	AP036015	AP036016 AP036016
BioProject ID	PRJNA1144671	PRJNA1144671
Run ID	SRR30222629	SRR30222628
Total reads (M bp)	996	2,947
Read N50 (bp)	22,880	21,194
No. of contigs	1	1
Coverage	×148	×348
Genome assembly size (bp)	66,98,676	84,51,396
No. of CDSs	5,712	7,794
G + C content (%)	45.7	44.8
No. of rRNAs	36	36
No. of tRNAs	104	109

The assembled genome of isolate WG01 was identified to be *Paenibacillus polysaccharolyticus* based on ANI of 97.76% to GCA_900102085.1 (7), and that of WG02 was inconclusive at the species level (ANI of 95.97% to GCA_013266765.1), according to DFAST Taxonomy assignment (6). The complete genome of *Paenibacillus polysaccharolyticus* WG01 had a total length of 6,698,676 bp, and that of *Paenibacillus* sp. WG02 was 8,451,396 bp, harboring 5,712 and 7,794 coding genes, respectively.

## Data Availability

The genome sequences reported here were deposited in DDBJ, and the raw reads were deposited in the Sequence Read Archive (SRA), under the accession numbers listed in Table 1. https://www.ncbi.nlm.nih.gov/bioproject/1144671 http://getentry.ddbj.nig.ac.jp/getentry/na/AP036015/ http://getentry.ddbj.nig.ac.jp/getentry/na/AP036016/
